# Deploying digital health tools within large, complex health systems: key considerations for adoption and implementation

**DOI:** 10.1038/s41746-022-00557-1

**Published:** 2022-01-27

**Authors:** Jayson S. Marwaha, Adam B. Landman, Gabriel A. Brat, Todd Dunn, William J. Gordon

**Affiliations:** 1 Department of Surgery, Beth Israel Deaconess Medical Center, Boston, MA USA; 2 Department of Biomedical Informatics, Harvard Medical School, Boston, MA USA; 3 Department of Emergency Medicine, Brigham and Women’s Hospital, Boston, MA USA; 4 Atrium Health, Charlotte, NC USA; 5 Department of Medicine, Brigham and Women’s Hospital, Boston, MA USA

**Keywords:** Health services, Health policy

## Abstract

In recent years, the number of digital health tools with the potential to significantly improve delivery of healthcare services has grown tremendously. However, the use of these tools in large, complex health systems remains comparatively limited. The adoption and implementation of digital health tools at an enterprise level is a challenge; few strategies exist to help tools cross the chasm from clinical validation to integration within the workflows of a large health system. Many previously proposed frameworks for digital health implementation are difficult to operationalize in these dynamic organizations. In this piece, we put forth nine dimensions along which clinically validated digital health tools should be examined by health systems prior to adoption, and propose strategies for selecting digital health tools and planning for implementation in this setting. By evaluating prospective tools along these dimensions, health systems can evaluate which existing digital health solutions are worthy of adoption, ensure they have sufficient resources for deployment and long-term use, and devise a strategic plan for implementation.

## Background

The digital health industry has seen rapid expansion over the past several years. Over the past decade, over 1200 US digital health companies have attracted a cumulative 33 billion USD in investment, from 1.1 billion USD in 2011 up to 14 billion USD in 2020^1^. The Food and Drug Administration (FDA) has also just recently begun approving digital therapeutics, and options for consumer-facing digital health tools are rapidly growing^2,3^.

While large health systems are increasingly looking to invest in digital health tools, the landscape of this field is very complex^4,5^. The market for health-system-based digital health tool development includes not only many start-up companies, but also several incumbents and entrenched players like large electronic health-record (EHR) vendors; there are innumerable regulatory-compliance considerations; and there are several unique barriers to the successful adoption of new digital health tools within these large, complex organizations^6,7^. There is no clear framework by which health systems can evaluate the realities of selecting, building, and deploying a digital health tool in a complex system^8^.

Many frameworks exist for clinical validation of these tools^3,9–11^, but few strategies describe how to integrate these validated tools in health systems; the literature on digital health implementation within large organizations is limited^12–14^. Existing frameworks for digital health implementation are difficult to operationalize for these dynamic organizations as they do not clearly define which stakeholders to involve, their roles, or the sequence of recommended steps^15^. The need to more critically evaluate this process is increasingly evident: the COVID-19 pandemic illustrated the value of many digital health tools^16^, but also revealed that there are adoption and deployment strategies that lead to minimal value creation, large amounts of frustration and inefficiency, and in some cases even patient harm^17^.

In this piece, we highlight the unique dimensions along which health systems should evaluate digital health tools to anticipate the potential benefits and challenges of adopting them. Key stakeholders who draw from experience in four large organizations—Brigham and Women’s Hospital (Boston, MA), Beth Israel Deaconess Medical Center (Boston, MA), Atrium Health (Charlotte, NC), and Intermountain Healthcare (Salt Lake City, UT)—convened to identify common lessons from implementation of digital health tools within these systems. In this piece, we define digital health tools as software applications developed for the purpose of improving healthcare services, such as providing clinical decision support or automating administrative or research processes within health systems^14^.

## What is the optimal product selection approach?

**Fig. 1:**
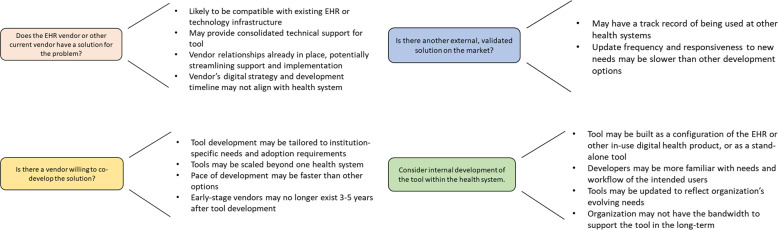
Approaches to digital health tool selection. Various digital health-product-selection approaches, and important considerations for each approach. We recommend investigating the viability of all four possibilities in parallel; the optimal approach will depend on the type of problem being addressed and characteristics of the health system.

Digital health tools can be built in a number of different ways (Fig. 1). Considering how a tool will be built (or has been built) may offer some insight into its strengths and weaknesses and can help a health system identify which product-selection approach is best in the context of the problem they are trying to solve. Depending on the needs and constraints of the institution, it may be best to purchase a complete product, configure an existing tool, or build a tool internally.

The advantages and limitations of each product-selection approach depend largely on the type of problem that the tool is intended to address, and the characteristics of the institution. Thus, an intimate understanding of the organization’s specific need or problem in context is essential before considering any tool or selection approach. If the tool is being built to address a site-specific problem unique to one health system, then an internally developed tool may be best. If the problem is universal, an externally built tool that has been validated elsewhere may be better. Internal custom development may also be preferable when there is no externally built tool currently available and a solution is needed immediately.

During the early months of the COVID-19 pandemic, US health systems leveraged many different development pathways to solve different types of problems. The need to rapidly expand telemedicine capacity was a pressure felt almost universally by health systems, so many quickly identified externally-built tools from third-party vendors^18^. Similarly, some health systems turned to existing external SMS-based or chatbot-based vendor solutions to help screen patients and direct them to the appropriate resources^19^. In contrast, each state enacted slightly different restrictions on postponing elective surgeries; since the criteria varied from state to state, many health systems turned to internal software-development teams to generate customized tools to comply with state rules. For example, to comply with Wisconsin-specific vaccine distribution and prioritization criteria, ThedaCare (a Wisconsin-based health system) turned to their EHR vendor to build a customized tool^18^. The Mass General Brigham health system (Boston, MA) internally developed a tool to comply with specific guidance from the Massachusetts Department of Public Health (MDPH) on screening visitors and employees for respiratory symptoms before entering healthcare facilities (Table 1)^20^.

**Table 1:** Key considerations for digital health tool adoption.

Key consideration	Description	Example 1: OR scheduler	Example 2: COVID pass
Product selection	Was the tool developed internally, by a third-party, by the health system’s existing EHR vendor, or through a private sector partnership?	The OR scheduling tool was developed as an additional feature by a development team at the hospital’s existing EHR vendor.	No tool that met this need was offered by the EHR or other existing vendor, so the tool was initially developed by an internal digital health team that used an existing application development tool supported by the organization. Given the need to scale a robust solution within a very large organization and the continued need for custom features, the internal team quickly moved to a custom.NET Framework application solution^20^.
Financial value	What framework does the tool leverage to generate financial value, and does it outweigh the costs associated with deployment and maintenance?	Use of this tool at other health systems has resulted in 10% increased OR utilization per day, resulting in the ability to perform an additional 10 procedures per week. The financial benefit of increased procedural volume outweighs the tool’s nominal cost of ongoing maintenance, which is already included in the health system’s payments to the EHR vendor.	Value was created by regulatory compliance with state-issued orders. The cost of deployment and maintenance was limited, as existing internal resources were re-deployed to focus on this product. COVIDPass was later used for vaccine and test scheduling, which generated financial ROI from automating the scheduling process.
Clinical value	Is there a clear, meaningful outcome metric consistent with the Quadruple Aim that would be improved by adopting this tool?	Prior literature shows that increased OR utilization rates at other health systems has resulted in higher rates of surgeon-reported satisfaction, lower surgeon burnout rates, and faster OR access for emergent procedures.	The impact of the tool was measured in terms of the number of symptomatic individuals successfully prevented from entering the facility, the fraction of individuals tested for COVID-19 within 14 days of a positive screen, and the fraction of individuals with a positive screen who truly had a COVID-19 infection^20,24^
Data assets	Does the health system have access to the data necessary for tool functionality? Is the tool interoperable with the health system’s IT infrastructure? Is training data needed to tune the tool to the local environment? Has data governance been established?	The tool uses a machine learning algorithm that must be trained on local data to predict procedure duration within this health system. The tool requires hourly OR schedule data updates, which the health system’s IT infrastructure can support. Procedure duration and schedule data sharing is covered under an existing DUA between the EHR vendor and hospital.	All COVID Pass user responses were stored in one central, secure database. The internal development team created an aggregate dashboard to share data with site operational leaders and Human Resources leaders daily.
Internal champion	Is there an advocate within the health system with the leverage and motivation to facilitate adoption and implementation?	The Chair of the Department of Surgery at this health system had been struggling to ensure that all surgeons have sufficient dedicated OR time, and was interested in using this tool to free up additional OR time. The head of Perioperative Services was tasked with training and soliciting feedback from schedulers.	The Associate Chief Medical Information Officer managed development and implementation of the COVID Pass tool.
Executive sponsors	Is there a senior executive-level advocate who will help support and pay for the tool’s adoption and deployment?	The health system’s CEO recognized the financial value of more efficiently utilizing OR time, and dedicated resources to adopting and deploying the tool. The CMIO ensured the tool met their interoperability and security standards.	The health system’s Chief Human Resources Officer was the executive sponsor and guided product strategy. The hospital’s CIO dedicated resources and oversaw overall development and deployment.
Institutional priorities	Does the tool align with institutional goals or aid in regulatory compliance?	Unutilized OR time has been estimated to currently cost this health system 35 USD per minute, and currently 20% of its OR time is unutilized.	Cases of COVID-19 in Massachusetts were increasing exponentially at the time and the hospital had an urgent need to ensure safety of patients and staff. The State of Massachusetts also issued orders requiring screening of staff and visitors prior to entering healthcare facilities.
Implementation	What IT, training, and workflow modification resources are needed for implementation?	The health system requested the EHR vendor to add this feature at their site. Perioperative staff were trained on how to enter daily OR schedules and new OR cases into this tool.	The development team engaged marketing and communications to create an easy URL and QR codes for the tool. The tool was also introduced as a smartphone app and distributed through the hospital’s employee app catalog. Kiosks were set up at hospital entrances to screen employees and visitors with the tool prior to entry.
Long-term operational home	Who will continue to provide technical support and quality assessment of the tool into the future?	The EHR vendor provided ongoing technical support. An OR leadership group within the Department of Perioperative Services was tasked with overseeing the tool, funding it in the long-term, and assigning business analysts to regularly assess OR utilization metrics.	COVID Pass remained under the ownership of the hospital’s in-house digital health development and adoption team, who continued to add features to the screening tool as the demands of the COVID-19 pandemic evolved.

Key considerations for health systems when evaluating digital health tools for adoption and implementation: two examples of each consideration are provided. Example 1 is of a health system seeking to increase OR utilization and evaluating a tool that is designed to efficiently schedule operating-room (OR) cases. Example 2 is of a health system at the beginning of the COVID-19 pandemic in March 2020, facing a need to rapidly begin screening patients, visitors, and employees for respiratory symptoms prior to entering the hospital^20^.

Internally built tools are also often built with a deeper understanding of the health system’s problem in context, and tailored to the IT configuration and security standards of the health system. Internally developed tools can also be frequently updated to reflect the specific needs of the organization. A drawback, however, is that an internal department may not have the bandwidth to troubleshoot or update the tool frequently or rapidly. While this drawback for internally developed tools is a strength of third-party tools, integration and interoperability with a health system’s existing IT infrastructure may be a challenge of third-party products. Further, external tools are often updated less frequently and have their own development priorities that may not match the needs of the organization. When evaluating a third-party tool, a health system must ask itself the following question: could a tool by an existing vendor of ours (e.g., our existing EHR vendor), or one that already exists internally, accomplish the same task? If so, these options may be more favorable to adopting a new third-party tool. Duplicative efforts should be avoided at this stage by identifying and repurposing existing tools within the health system that may already address the same problem.

Another advantage of externally built tools is that they may already be in use at other health systems. Health systems should seek out such case studies or references when evaluating third-party tools. Assessing the experience of these other health systems in using this tool may reveal valuable evidence for or against the adoption of the tool. For this reason, there is an important distinction between digital health tools built by the EHR vendor as existing products versus those built as site-specific configurations. Products offered by the EHR vendor may be more likely to have a track record of deployment and use at other health systems previously, which can be used to assess the tool’s value. In some cases, health systems use varying configurations of the tool; this enables some EHR vendors to offer libraries in which other health-system customers can share their tool configurations. For example, in the case of a health system seeking to address a common problem such as poor operating-room (OR) utilization, their EHR vendor may offer an OR-scheduling tool as an optional add-on (Table 1).

Increasingly, an additional model of product development is also being adopted by large academic medical centers in which they form private-sector partnerships to codevelop digital health tools^14,21^. This approach has many advantages for health systems, including the ability to tailor tools for ease of adoption and institution-specific needs, the ability to scale the tool beyond one health system, and the ability to generate nonclinical revenue through IP licensing^4^. It may be advantageous for vendors as well, as it increases the likelihood of adoption of the product they are codeveloping.

Given the complementary opportunities and challenges associated with each method of digital health-tool selection, we suggest giving four methods full and equal consideration before selecting one. Once there is a deep understanding of the need that the health system is trying to meet, first consider if the organization’s EHR vendor or vendor for an existing digital health tool offers a solution. Second, consider if there is another external, validated solution on the market. Third, consider identifying a vendor who is willing to codevelop the solution. Finally, also consider internal custom development of the desired tool. In Fig. 1, we highlight considerations for each of these approaches. The ideal product-selection approach may vary by a health system’s specific needs.

## Is there a clear demonstration of ROI?

While CMS has not yet issued clear guidance on reimbursement for use of digital health tools, there are a number of other ways use of these tools can generate ROI^2^; understanding the framework by which the tool generates this value is important. ROI in health systems is driven by a number of factors, and it should be clear which factor the tool addresses. Some frameworks and examples by which tools can generate financial ROI are shown in Box 1. For example, a tool that increases OR utilization through more efficient scheduling of cases would generate financial value in a fee-for-service model by increasing procedural volume.

In considering the financial value of the tool, it is also important to measure how much the tool costs to acquire and maintain and who will be paying for it. A tool may have a time-limited capital expense associated with it, or an operational expense that incurs costs in perpetuity, or in most cases, both. In adopting a digital health tool, there is often not only an upfront cost associated with implementation, but also ongoing costs and staffing requirements associated with support, maintenance, and hosting. Once these upfront and long-term costs are estimated, it is also important to consider whether there is an existing line item in the health system’s budget for the tool, or if a new source of funding is required.

Finally, the overall business plan of the tool should be examined by comparing the financial value it generates against costs associated with long-term maintenance. Once the tool has been implemented, it will likely continue to incur operational expenses and also require support personnel, computer and storage space, and project managers to oversee updates and maintenance. These responsibilities may be distributed in various ways across internal staff at the health system, the vendor, or even third parties to whom the health system can outsource support tasks.

Box 1. Return on investment (ROI) frameworks Common frameworks by which digital health tools can demonstrate financial return on investment (ROI).

**Table:** 

Framework	Details
Fee-for-service	• Does the tool increase procedural volume? • Does it enable providers to see more patients?
Value-based care	• Does the tool reduce the cost of care for certain conditions or populations? • Does it reduce total medical expenses for certain populations? • Does it improve a specific clinical outcome?
Regulatory compliance	• Does the tool help the health system comply with regulations that are tied to financial incentives or penalties?

## Is there a clear demonstration of clinical value?

There are a number of clinical dimensions along which to evaluate a digital health tool’s value and risks^22^. Identifying a meaningful clear outcome metric that would be improved by adoption of the tool in the short term is essential when considering adoption. A meaningful metric is one that affects an aspect of the Institute for Healthcare Improvement (IHI)’s Quadruple Aim: improving the health of populations, enhancing the experience of care for individuals, reducing the per-capita cost of health care, and improving the experience of clinicians and staff^23^. Examples of such metrics include provider burnout, time savings, patient outcomes, and patient satisfaction. In the case of externally developed tools, the vendor sometimes may be able to provide customer case studies or scientific evidence showing that the use of their product is associated with improvements in some meaningful outcome of interest. Measuring a tool’s direct impact on a granular outcome—rather than a more high-level one—may be a better way to capture its value. For example, it may be difficult to measure a tool’s “total provider time saved,” and easier instead to measure its “reduction in number of unnecessary consultations.” For example, the Mass General Brigham health system measured the impact of their COVID Pass screening tool in terms of the number of symptomatic individuals successfully prevented from entering the facility, the fraction of individuals tested for COVID-19 within 14 days of a positive screen, and the fraction of individuals with a positive screen who truly had a COVID-19 infection^20,24^.

A tool may be designed to improve a specific outcome, but whether or not it is actually capable of achieving this goal in clinical practice ultimately dictates its true clinical value. A digital health tool that is useful in practice is one that is able to trigger a specific workflow: the insight that the tool provides must be translated into an actual human intervention in order to realize the tool’s value^25^. Prior work suggests that predictive models have the capacity to move the needle on clinical outcomes when their output is coupled with tailored human interventions^26^. Thus, both the tool’s target-outcome metric and its ability to affect this outcome metric must be considered.

Off-the-shelf tools sometimes have a track record of use at other health systems that may offer insight into their clinical value. However, in the case of tools with no prior use history (e.g., those that have been internally developed by the organization), a limited pilot among a small sample of the total intended user population may help the organization assess if the new technology actually affects the desired outcome metric or not. Validated resources for designing effective pilots are described elsewhere^14^.

## What data assets are required for product functionality?

Digital health tools are typically informed by one or more sources of data when generating outputs such as prognostic or diagnostic predictions, administrative tasks, or documentation recommendations. Common sources of structured and unstructured data include the EHR, scheduling or administrative systems, and administrative claims and billing systems. The health system must ensure that the new tool has access to the appropriate stream of data at the appropriate interval (e.g., real time, weekly, or monthly) for full functionality. The tool must also be fully embedded within the health system’s information ecosystem: it should be able to send and retrieve data from other applications as needed for functionality and accessibility. Encouragingly, a growing number of tools are being built that directly connect to EHRs and use standards-based application programming interfaces (APIs) such as fast healthcare interoperability resources (FHIR)^27^. Some organizations have also begun curating these products based on their interoperability with existing systems^28^. Ensuring that the tool shares a common data standard with other tools in the organization is essential. In addition, for off-the-shelf tools built by external vendors that are implementing machine-learning algorithms, local training data may be required to tune the model’s hyperparameters to the new environment it will be deployed within, prior to deployment. Finally, in order to share these data with a third-party vendor, the organization must ensure that there is some data-governance structure in place, including a data-use agreement (DUA) that allows the vendor to access the minimal necessary data for tool functionality. Importantly, the DUA should specify how patient-level data are to be protected (e.g., a DUA might specify that any data shared with the vendor must be anonymized immediately prior to use), and should define what—if any—secondary use of the data is permitted. The Department of Health and Human Services (HHS) has proposed best practices for DUAs^29^. In the case of our OR scheduling tool example, local training data were needed to inform the tool of how long various procedures at this health system typically take. Fortunately, a previously established DUA between the EHR vendor and the hospital covered this data sharing (Table 1).

## Is there an internal champion?

An internal champion is someone with an intimate understanding of how the tool meets a specific need of the organization, the bandwidth and motivation to orchestrate adoption of the tool (including IT integration and personnel training), the leverage to encourage people to use it, and is similar enough to the typical user to envision how it should be integrated into existing workflows. The internal champion must be able to engage a representative sample of prospective users (including clinical, management, and administrative users) prior to, during, and after implementation, who can provide continual feedback on both the new product and new process created by the product^30,31^. To improve the likelihood of successful implementation of health IT products, prior literature supports obtaining feedback from representative samples of all distinct groups that interact with the tool, such as nurses and physicians, and finding inclusive solutions that address each group’s needs^32–34^. Important feedback on the product may include comments on the interpretability, reliability, and usability of its output or the algorithm it uses; feedback on the process may comment on how disruptive or time-consuming the workflows triggered by the tool’s various outputs are.

We recommend identifying an internal champion that resembles a potential user as closely as possible. For example, an OR-scheduling tool should have an OR-scheduling administrative staff member as its internal champion. Nurses, physicians, advanced practice providers (APPs), physical/occupational therapists, and administrative staff are all examples of possible internal champions as they are in a unique position to identify digital health tools that are suitable for adoption, have an intimate knowledge of the health system’s daily processes, and have the leverage to encourage their colleagues to use the tool and provide feedback. In the case of digital health tools that are being implemented for the purposes of research or data collection, having an internal research champion—such as the principal investigator of a research laboratory within the organization—is also important.

## Are there executive sponsors?

The adoption and implementation of digital health tools must be considered from the perspective of two executive-level strategies. One is the organization’s digital strategy: ensuring that new tools are compatible with the organization’s IT infrastructure and platforms, information-security requirements, and more. The other is the organization’s broader healthcare-delivery strategy: ensuring that new tools align with the general purpose, direction, and needs of the health system. To address these two strategies, two or more executive sponsors may be needed.

A senior health-system executive such as the chief executive officer, chief medical officer, chief nursing officer, or senior vice president of clinical services may be in a good position to ensure that the tool aligns with the organization’s healthcare-delivery strategy, and also that the tool’s business plan justifies its adoption. This executive sponsor may also be able to support the tool’s adoption—even if they do not engage directly with the tool nor resemble the tool’s typical user—by providing any necessary resources identified by the internal champion. The second executive sponsor should address the organization’s digital strategy. A chief digital officer or chief medical informatics officer may be able to ensure that the solution being selected will adhere to the organization’s digital strategy for interoperability and security.

## Does the tool align with institutional priorities?

A number of existing digital health tools have the ability to demonstrate ROI and clinical value. Only the ones that align with institutional priorities, however, are likely to attract the momentum necessary for widespread adoption and deployment. A tool—and the outcomes it targets—must align with the goals and strategy of the health system’s executive leadership, such as expanding to new markets or addressing specific conditions. An understanding of these goals can help identify tools that are most likely to gain the support of executive leadership. As discussed in the prior section, a key role of the executive sponsor should be to ensure this institutional alignment of priorities.

Regulatory compliance is another way a tool can meet institutional priorities. Compliance with upcoming legislative deadlines is often a catalyst for adoption of tools that help health systems comply. For example, the ONC’s Final Rule, which requires increased electronic health information (EHI) sharing between health systems and patients, will likely catalyze adoption of new digital health tools that enable this sharing among health systems as they race to become compliant with upcoming deadlines. In the case of COVID Pass, the institution’s urgent need to limit unnecessary COVID-19 exposures to patients and staff—as well as issues ordered by the State of Massachusetts—prompted them to develop and deploy the tool within two weeks of conception.

## What resources are required for implementation?

Once a tool has been selected or built, the resources necessary for implementation should be identified. Whether a tool’s implementation will be resource- and effort-intensive or not can likely be estimated based on the scope of its domain and its relationship to existing workflows—namely, how many workflows it affects and whether it burdens or improves these workflows, as described recently by Morse et al.^8^. For example, a general inpatient-deterioration prediction tool may burden the workflow of multiple care teams by giving them information that they would normally not evaluate and are unsure how to respond to. Such a model would also require large numbers of staff—primary teams, consulting teams, and nurses—to be trained on how to react to its output. In contrast, an OR-scheduling tool designed for the very narrow purpose of scheduling procedures in a time-efficient manner may only affect the workflow of perioperative schedulers, increase the efficiency of the scheduling workflow, and only require scheduling staff to be trained in its use.

We recommend identifying the resources necessary to address five issues: (1) training, (2) IT integration, (3) information security, (4) human capital investment, and (5) adapting existing real-world workflows and EHR workflows to incorporate the new tool. Important considerations for each of these five issues are listed in Box 2. The US Office of the National Coordinator for Health Information Technology (ONC) has published guides that offer further detail on ensuring information security, interoperability, and preparing the necessary hardware and software infrastructure for health IT implementation^35,36^. Other research groups have offered further detail on preparing for training and adapting workflows^32,37^.

Given these multiple simultaneous considerations and the overall complexity of implementation, there is some risk of developing incomplete implementation plans or losing momentum while planning the implementation process. Some institutions have developed process maps to help standardize the pilot and clinical validation processes for tools they are considering implementing—a similar methodical approach to implementation may be beneficial^14,38^. We suggest using the nine key considerations outlined in this piece as a checklist to standardize digital health-tool selection and to prepare for implementation. Health systems may customize the considerations listed in this piece to their organization’s needs, to ensure that all aspects of implementation are considered for each tool, that they are considered in the right order, and that teams responsible for implementation are able to promptly connect with appropriate contacts in each area to maintain momentum.

Box 2. Identifying resources for implementation Important considerations when identifying what resources are required for digital health tool implementation.

**Table:** 

Domain	Considerations
Training	• Through what medium should the training be delivered? • What types of training materials must be prepared?
IT integration	• What are the specific integration requirements for the product itself? • What permissions are required to integrate the tool into the health system’s existing IT infrastructure?
Information security	• What are the organization’s standards for information security, and does the tool meet these standards? • Are there any security or privacy issues that need to be mitigated prior to implementation?
Human-capital investment	• What are the personnel and technical expertize requirements for training, integration, and information-security activities?
Adapting existing real-world and EHR workflows	• How will provider and staff responsibilities change after deploying the tool? • What components of the EHR interface need to be changed to adapt to this new tool?

## Does the tool have a long-term operational home?

Digital health tools in large health systems are sometimes viewed as innovation projects and receive short-term funding for adoption and deployment. However, at some point, these projects must graduate to an operational home. It is important to identify who will be responsible for “owning” the tool’s functionality, financial sustainability, and clinical impact in the long-term before it is adopted.

One dimension of long-term ownership that should be considered is whether the tool will have a “technical owner” who has the ability to provide technical support and maintenance into the future. For internally developed tools, there must be capacity within the health system for informatics staff to identify, understand, and develop solutions to future issues with the tool; there must also be capacity for technical IT staff to implement these proposed solutions, host the tool, and handle other technical issues. For externally developed tools, technical support contracts with vendors should be maintained. Contingency plans should be made as well: what if the vendor gets acquired or goes out of business and can no longer offer ongoing technical support? As more workflows and functions in healthcare are digitized, health systems will likely need to expand their own technical teams to support these tools.

Another dimension of long-term ownership that should be considered is who the tool’s “business owner” will be. An operational group should be responsible for overseeing the tool, continuing to fund it, and performing ongoing quality assessments: the tool should be continually reexamined to ensure it is delivering its intended value, and iteratively refined if it is not. This may require assigning specific project managers or business analysts to this long-term task. COVID Pass remained under the ownership of the in-house digital health development and adoption team, who continued to solicit feedback from Human Resources and Occupational Health on new features such as informing users of COVID-19 clinical trials and enabling scheduling of vaccine and testing appointments. The OR-scheduling tool was placed under the ownership of the Department of Perioperative Services, whose responsibilities included overseeing the tool, incorporating it in their budget, and assigning business analysts to regularly assess OR-utilization metrics (Table 1).

## Conclusion

Large health systems face unique, complex challenges in adopting, implementing, and operationalizing digital health tools, which stand in the way of significant potential improvements in healthcare services. In this piece, we identify nine key considerations to help these organizations identify and implement digital health tools strategically. By evaluating prospective tools along the dimensions described in this piece and summarized in this table—the product-selection approach, the ROI and clinical value, internal champions and executive sponsors, data assets required for functionality, alignment with institutional priorities, requirements for implementation, and long-term operations—health systems can decide on how best to select or develop a digital health solution, evaluate whether an existing tool is worthy of adoption, ensure they have sufficient resources for deployment and long-term use, and devise a plan for implementation.
